# Systematic Review and Meta-Analysis of Tuina as an Adjuvant Therapy for Functional Constipation in Children

**DOI:** 10.3390/healthcare14070901

**Published:** 2026-03-31

**Authors:** Qingping Shi, Beiyan Chen, Shuang Gao, Mingli Shen, Jieru Han

**Affiliations:** School of Basic Medical Sciences, Heilongjiang University of Chinese Medicine, Harbin 150040, China; sqp17326705862@163.com (Q.S.); chenbeiyanabc@163.com (B.C.); gaoshuangacc@163.com (S.G.); 18309474893@163.com (M.S.)

**Keywords:** functional constipation, child, tuina, adjuvant therapy

## Abstract

**Background**: Functional constipation (FC) in children is characterized by infrequent bowel movements and straining or painful defecation, often accompanied by abdominal pain and fecal incontinence. Typically, in cases without organic pathology, it is diagnosed as this disease. When diagnosed according to Rome IV criteria, the global prevalence of this condition is 14.4%. Additionally, one study reported that in the UK, one in seven adults and one in three children suffer from constipation. In just one year (2018–2019), the National Health Service incurred £168 million in costs related to constipation, with over 175,000 hospital days consumed and numbers continuing to rise. Massage therapy is viewed as a promising treatment modality, though systematic evaluation evidence for its use in combination therapies remains insufficient. **Research Objective**: This study aims to systematically evaluate the efficacy and safety of tuina massage as an adjunct to conventional treatment for pediatric functional constipation through meta-analysis. **Materials and Methods**: Eight databases were searched to collect relevant randomized controlled trials (RCTs) from their inception to 23 January 2026. Primary outcomes included clinical total effective rate and BSFS score. Secondary outcomes comprised adverse reaction incidence, recurrence rate, defecation difficulty, and improvement in bowel movement frequency. Quality assessment was performed using the Cochrane Risk of Bias tool (RoB 2). Meta-analysis was conducted using RevMan 5.4 and Stata 13 software. **Results:** A total of 16 RCTs involving 1387 pediatric patients were included. Meta-analysis revealed that compared with conventional therapy alone, the combination of Tuina and conventional therapy significantly improved the overall clinical response rate (RR = 1.18, 95% CI 1.10 to 1.25, *p* < 0.00001; Tau^2^ = 0.01), improved the Bristol Stool Form Scale (BSFS) score (MD = 0.55, 95% CI 0.32 to 0.78, *p* < 0.00001; Tau^2^ = 0.05), and reduced the defecation difficulty score (MD = −1.36, 95% CI −1.75 to −0.98, *p* < 0.00001; Tau^2^ = 0.18). However, substantial heterogeneity was observed across these outcomes (I^2^ = 75%, 71%, and 96%, respectively). The 95% prediction intervals crossed the null value for all three primary outcomes (treatment success rate: 0.95–1.47; BSFS score: −0.25 to 1.35; defecation difficulty score: –2.85 to 0.13), indicating that the true effect may vary substantially across future study settings. Regarding recurrence, Tuina-assisted therapy resulted in a lower recurrence rate compared to conventional therapy alone (RR = 0.27, 95% CI 0.16 to 0.47, *p* < 0.00001). While improvements in weekly bowel movement frequency were reported, they could not be confirmed due to insufficient sample size. It remains unclear whether Tuina can mitigate adverse effects associated with control group treatments. **Conclusions:** Current evidence suggests that tuina as an adjunct to conventional treatment may offer improvements in treatment success, stool consistency, defecation difficulty, and recurrence rates in children with functional constipation. However, given the substantial heterogeneity (I^2^ up to 96%), wide prediction intervals that crossed the null value for all three primary outcomes, methodological limitations of the included studies (e.g., lack of blinding, unclear randomization), and short follow-up periods (most ≤2 months), these findings should be interpreted as exploratory rather than definitive. Evidence on weekly stool frequency and adverse reactions remains inconclusive. High-quality, long-term trials with standardized outcome measures and rigorous blinding are needed to validate these preliminary findings.

## 1. Introduction

Functional constipation is initially managed with standard therapeutic approaches, including pharmacological treatment with laxatives (e.g., polyethylene glycol, lactulose), increased fluid intake, and a high-fiber diet rich in fruits [1]. Despite these conventional measures, symptom control remains suboptimal in a subset of patients, prompting interest in complementary therapies.

In pediatric and adolescent populations, the vast majority (90%) of constipation cases are functional, while a minority (10%) are organic [2]. Functional constipation (FC) in children is characterized by infrequent bowel movements and straining or pain during defecation, and is often accompanied by abdominal pain and fecal incontinence. Generally absent in organic lesions, it is thus diagnosed as this condition [3]. According to modern medical theory, FC arises from a combination of factors including fecal retention behavior, anorectal dysfunction, dietary influences, physical activity levels, genetic predisposition, and psychological issues [4]. Diagnosed according to Rome IV criteria, the global prevalence of this condition is 14.4%. By continent, Africa has the highest constipation prevalence (31.4%), followed by the Americas, Europe, and Asia [5]. Functional constipation imposes a serious financial burden on healthcare systems in many countries. For example, one study reported that in the UK, one in seven adults and one in three children suffer from constipation. In just one year (2018–2019), the National Health Service incurred £168 million in costs related to constipation, with over 175,000 hospital days consumed and numbers continuing to rise. Massage therapy is viewed as a promising treatment modality, though systematic evaluation evidence for its use in combination therapies remains insufficient [6]. Another study estimated that childhood constipation in the United States incurs an additional annual cost of approximately $3.9 billion [7].

The North American Society for Pediatric Gastroenterology, Hepatology and Nutrition (NASPGHAN) guidelines recommend initial treatment followed by maintenance therapy, utilizing laxatives, education, and other interventions [1]. One study reported that despite medical interventions, a significant proportion of patients continued to experience symptoms. In secondary and tertiary care settings, 40% of treated children still had symptoms after 6 to 12 months [8]. Studies with 5- to 10-year follow-ups reported that 50% of children recovered after 5 years, and 80% recovered after 10 years [1]. However, symptoms may persist into adolescence or adulthood for the remaining children. Despite ongoing laxative therapy, constipation issues persist [9,10]. Long-term medication use carries potential side effects including fecal incontinence, abdominal pain, nausea, bloating, and electrolyte imbalances [11].

Massage therapy for constipation may work by increasing vagus nerve activity via its branches innervating the gastrointestinal tract, thereby enhancing gastrointestinal motility [12,13]. Specific techniques include kneading, pressing, and circular movements applied to the lower abdomen, targeting areas such as the descending and sigmoid colon to promote fecal propulsion. In traditional Chinese medicine, Tuina (massage) incorporates acupoint stimulation—such as at Tianshu (ST25) and Dachangshu (BL25)—which has been shown to improve intestinal motility in rats [14,15]. These mechanisms—ranging from direct mechanical effects to reflex-mediated and neuromodulatory influences—collectively underpin the therapeutic potential of massage for relieving constipation.

A study on non-pharmacological treatments for pediatric functional constipation suggests massage therapy as a promising adjunctive approach [16]. Among traditional Chinese medical therapies, herbal medicine often faces poor compliance in young children due to taste and other factors [17]. Similarly, acupuncture, being invasive and sometimes misunderstood by families, also suffers from low compliance [17]. In contrast, massage therapy, as a non-invasive procedure, is generally well-accepted by both children and parents.

However, whether massage therapy as an adjunct to conventional treatment yields superior outcomes for pediatric functional constipation remains unclear. This systematic review and meta-analysis aims to evaluate relevant randomized controlled trials (RCTs) to determine whether Tuina, as an adjunct to conventional therapy, improves functional constipation in children compared to conventional therapy alone. Specific objectives include evaluating its impact on primary outcomes (stool consistency and treatment success) and secondary outcomes (recurrence rate, stool difficulty score, adverse reactions, and weekly bowel movement frequency) in order to synthesize evidence and support clinical decision-making.

## 2. Materials and Methods

Adhering to the PRISMA guidelines [18], this study ensured rigorous reporting; the review’s protocol was prospectively registered on PROSPERO (Registration number: CRD420261301080): https://www.crd.york.ac.uk/PROSPERO/recorddashboard (accessed on 5 February 2026).

### 2.1. Literature Sources

Potential eligible studies were identified by searching the following databases: China National Knowledge Infrastructure (CNKI), WanFang Database, VIP Database, SinoMed (CBM), the Cochrane Library, PubMed, EMBASE, and Web of Science.

### 2.2. Search Strategy

To conduct a comprehensive search, studies published up to 23 January 2026 were investigated, with no language restrictions. When searching English databases, the following combinations of English terms were used: “Functional constipation” OR “Constipation” OR “Colonic Inertia” OR “Dyschezia”, along with “pediatric” OR “adolescent” OR “child” OR “children” OR “kid” OR “youngster”, and ‘Tuina’ OR “massage”. The specific search formula is shown in File S1.

### 2.3. Inclusion Criteria

(1) Participants: All participants needed to meet the diagnostic criteria for functional constipation in children [19,20,21,22,23,24], with an age of onset between 1 and 18 years.
(2) Interventions: Participants in the experimental groups were administered Tuina therapy in combination with conventional treatment, consisting primarily of laxatives and/or probiotics. The control groups received conventional treatment alone. Apart from Tuina, all other treatments needed to be identical between groups.
(3) Study Design: Randomized controlled trials (RCTs) were included, regardless of whether allocation concealment or blinding is implemented.
(4) Outcomes: Primary outcomes: Overall treatment efficacy rate and Bristol Stool Form Scale (BSFS) score. Secondary outcomes: Adverse reactions, recurrence rate, severity of defecation difficulty, and post-treatment defecation frequency.
(5) We included only two-arm randomized controlled trials.

### 2.4. Exclusion Criteria

(1) Non-randomized controlled trials.
(2) Studies where the intervention was not Tuina therapy combined with conventional treatment alone.
(3) Literature with missing primary or secondary outcome data.
(4) Duplicate publications, reviews, or studies with incomplete data for which the full text was unavailable.
(5) Constipation caused by organic diseases/pathology.

### 2.5. Outcomes

**Primary Outcomes:**

(1) Bristol Stool Form Scale (BSFS) Score: Assessment was based on the Bristol Stool Form Scale, which classifies stools into seven types (I to VII) corresponding to scores of 0 to 6, respectively [25]. Statistical analysis was performed on the BSFS scores.
(2) Overall Effectiveness: Efficacy assessment was conducted according to the guidelines outlined in the Guiding Principles for Clinical Research of New Traditional Chinese Medicine Drugs [26] and the Criteria of Diagnosis and Therapeutic Effect of Diseases and Syndromes in Traditional Chinese Medicine [22]. Both evaluation methods focused on symptom relief assessed by evaluating bowel movement frequency, stool consistency, abdominal distension, and abdominal pain.
  In the included studies, despite minor variations in wording, the definition of “overall efficacy” consistently centered on these core clinical indicators.
  For example:
  - Markedly effective was defined as resolution of constipation symptoms with normalization of stool consistency (typically corresponding to a symptom score reduction >70%), or achieving smooth bowel movements with softened stools within 2 days.
  - Effective was defined as improvement in symptoms and stool consistency, with bowel movements occurring within 3 days or a 30–70% reduction in symptom scores.
  - Ineffective was defined as no improvement or bowel movements occurring only after one week.
  Methodological considerations regarding this outcome:
  Several methodological considerations should be noted regarding the overall effectiveness outcome. First, this outcome is inherently subjective, as it relies on clinician judgment based on a composite of symptom indicators rather than a single objective measurement. Second, although the core criteria were similar across studies, minor variations in definition (e.g., exact symptom score thresholds) and the lack of standardized operationalization introduce cross-study heterogeneity, potentially affecting the comparability of the pooled results. Third, the composite nature of this outcome does not allow differentiation of which specific symptom domains (e.g., stool frequency) contributed most to the treatment response. These considerations are important when interpreting the findings derived from this outcome.

**Secondary Outcomes:**

These included the incidence of adverse events, recurrence rate, severity of defecation difficulty, and weekly stool frequency.

### 2.6. Data Selection and Extraction

First, all literature was searched using EndNote software (version 21, Thomson Reuters, New York, NY, USA). Subsequently, two reviewers (Shi Qingping and Chen Beiyan) independently searched the aforementioned databases and listed all article titles. Based on the inclusion criteria, papers that did not meet the requirements were preliminarily excluded by reviewing titles and abstracts. Subsequently, articles underwent further screening based on content; those failing to meet requirements were excluded. Disputed content was resolved through communication with the corresponding author (Han Jieru). Data extraction included: first author, publication date, number of cases in experimental and control groups with childhood FC, specific details of the intervention, intervention duration, outcome measures, and data.

### 2.7. Quality Assessment

Two reviewers (Shi Qingping and Chen Beiyan) assessed the risk of bias in included studies using the Cochrane Risk of Bias tool RoB2 [27]. The risk of bias was assessed across the following domains: random sequence generation, allocation concealment, blinding of investigators and participants, blinding of outcome assessment, completeness of outcome data, selective reporting, and other potential biases [27]. Disagreements between reviewers were resolved through discussion. A rating of “low risk” indicated that the item was sufficiently informative. “High risk” signified inadequate or abnormal descriptions of methods or treatment protocols. “Unclear risk of bias” meant no description of methods or treatment protocols was provided. Any disagreements between assessors (Shi Qingping and Chen Beiyan) were resolved through consultation with the corresponding author (Han Jieru).

### 2.8. Statistical Analysis

The statistical analysis was performed using Review Manager (RevMan), version 5.4. For dichotomous outcomes, we calculated the relative risk (RR) and 95% confidence interval (CI). Continuous outcomes were pooled as the mean difference (MD) with 95% CI when measurement units were consistent across studies; the standardized mean difference (SMD) with 95% CI was applied when units differed.

Heterogeneity was assessed using the I^2^ statistic. A fixed-effects model was applied when I^2^ was less than 50%. When I^2^ exceeded 50%, indicating significant heterogeneity, the potential sources were investigated. First, the accuracy of the original data and data extraction process was verified. Subsequently, if heterogeneity could be attributed to factors such as race, dosage, randomization method, or allocation concealment, subgroup analyses were conducted. Sensitivity analyses were undertaken to identify sources of heterogeneity. Following this, if the heterogeneity could not be sufficiently explained, a random-effects model was deemed appropriate and subsequently applied.

Publication bias was assessed using funnel plots and Egger’s test, performed with Stata software. In accordance with methodological guidelines, these tests are typically meaningful when at least 10 studies are included, as it is difficult to detect asymmetry or determine its cause with fewer studies.

## 3. Results

### 3.1. Literature Search Results

**Figure 1:**
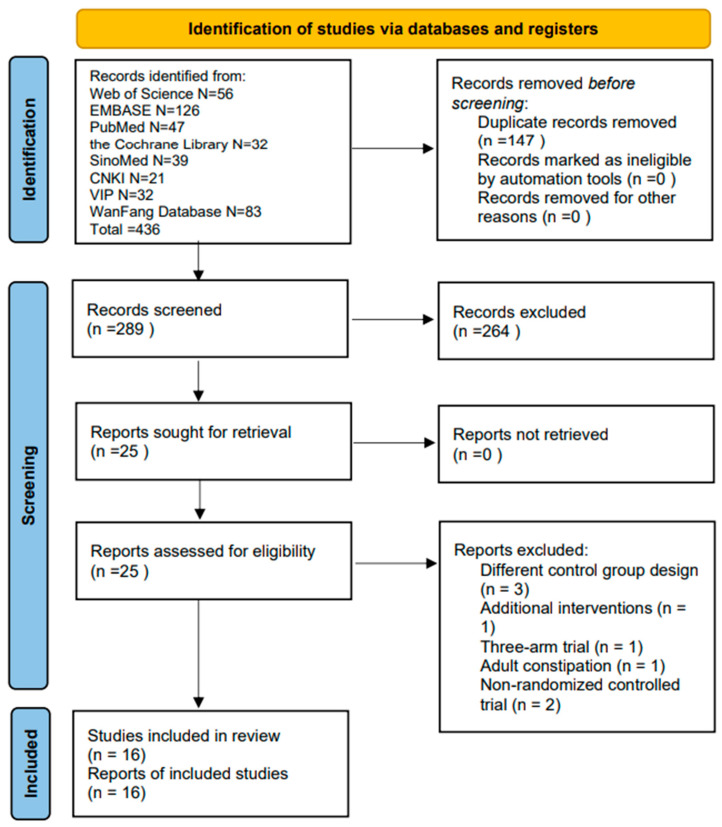
Inclusion process and results of the relevant articles.

A total of 436 records were identified from eight electronic databases. The initial search yielded 289 unique records after deduplication. Screening of titles and abstracts resulted in the exclusion of 264 records for the following reasons: 2 retraction notices, 32 with different control group designs, 7 non-randomized controlled trials, 2 three-arm trials, 6 with unavailable full texts, 52 involving interventions other than Tuina (massage), 25 reviews, and 139 studies unrelated to the condition. Full-text assessment of the remaining 25 articles led to the exclusion of 8 records (3 due to different control group design, 1 for having additional interventions, 1 three-arm trial, 1 on adult constipation, and 2 non-RCTs). Finally, 16 studies were included for analysis (see Figure 1).

### 3.2. Study Characteristics

**Figure 2:**
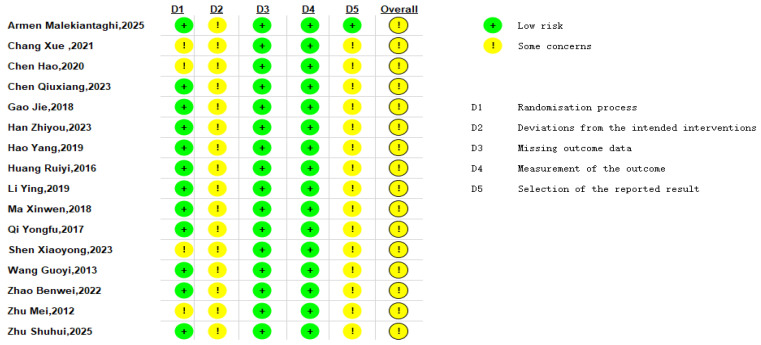
Results of Risk of Bias Assessment [28,29,30,31,32,33,34,35,36,37,38,39,40,41,42,43]. D1: Bias arising from the randomization process, D2: Bias due to deviations from intended interventions. D3: Bias due to missing outcome data. D4: Bias in measurement of the outcome. D5: Bias in selection of the reported result. Green indicates low risk. Yellow indicates some concerns.

**Figure 3:**
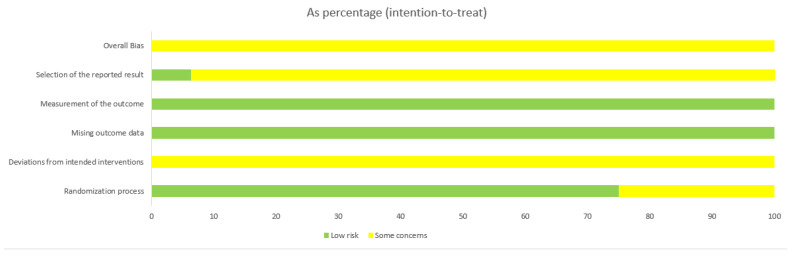
Results of Risk of Bias Assessment. Green indicates low risk. Yellow indicates some concerns.

A total of 1387 pediatric patients from 16 randomized controlled trials (RCTs) were included in the final analysis (Table 1). The publication dates of these trials ranged from 2012 to 2025. Studies were published in China and Iran. Sample sizes ranged from 50 to 120 participants. The intervention in the experimental group was massage combined with conventional treatment, while the control group received conventional treatment alone. The treatment duration ranged from 10 days to 1 month, with 3 studies failing to report the treatment period [28,29,30]. Three studies reported and compared the incidence of adverse reactions [30,31,32]. The primary outcome of treatment efficacy was reported in all included studies. Randomization methods were employed across all articles, though specific techniques were detailed in only five [29,30,32,33,34] (random number generator software and random number tables). Risk of bias assessments for included studies are shown in Figure 2 and Figure 3. We also performed a GRADE assessment of the primary outcomes of this studies, as detailed in Table 2. Baseline characteristics were consistent across 1387 patients, with no significant differences observed before intervention (see Table 1).

### 3.3. Effect Evaluation

#### 3.3.1. Effectiveness

**Figure 4:**
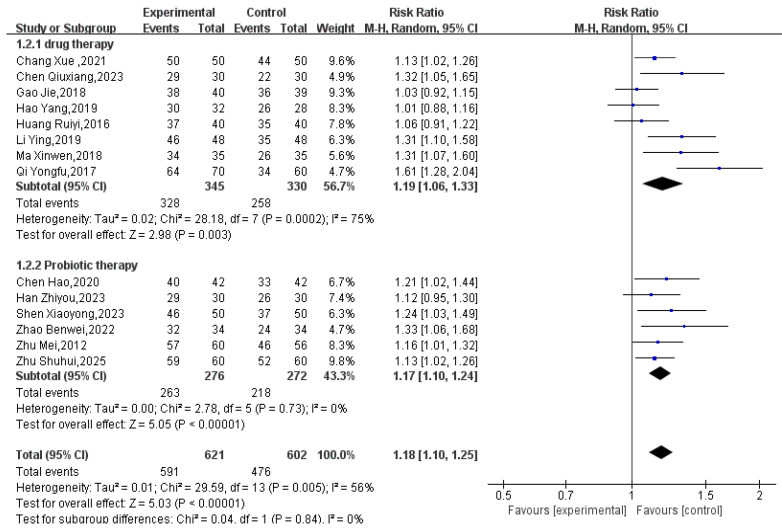
Image of Massage Adjuvant Therapy VS Conventional Treatment Regarding Efficacy [28,29,30,31,32,34,35,36,37,38,39,40,42,43].

**Figure 5:**
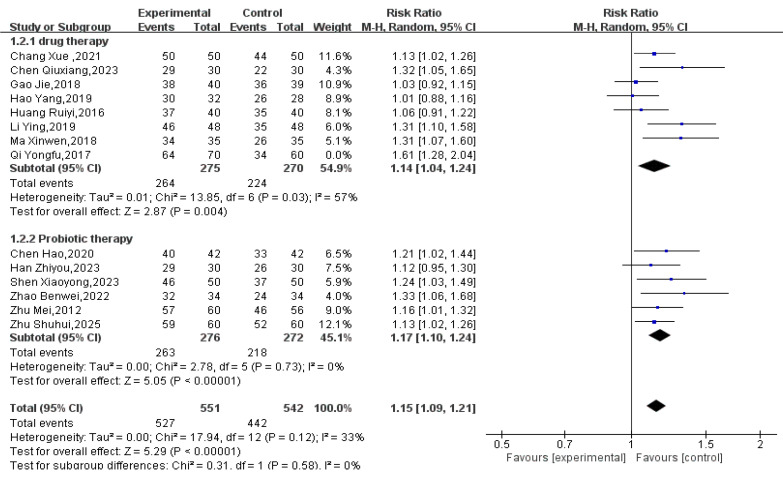
Figure Adjusted for Detected Heterogeneity [28,29,30,31,32,34,35,36,37,38,39,40,42,43].

A total of 14 RCTs involving 1223 participants (621 in treatment groups, 602 in control groups) reported efficacy data. The meta-analysis showed that Tuina as an adjunctive therapy was more effective than conventional treatment alone for pediatric FC (RR = 1.18, 95% CI 1.10 to 1.25, *p* < 0.00001; Tau^2^ = 0.01; see Figure 4). However, substantial heterogeneity was observed (I^2^ = 75%). The 95% prediction interval for the overall analysis ranged from 0.95 to 1.47, which crossed the null value (RR = 1.0), indicating that the true effect may vary substantially across future study settings. A sensitivity analysis excluding one study [29] reduced the heterogeneity to I^2^ = 57% (Tau^2^ = 0.00). In this sensitivity analysis, the prediction interval narrowed to 1.12 to 1.18 and no longer crossed the null value, suggesting consistent effects across the remaining studies. Pre-specified subgroup analysis based on type of control (probiotics vs. laxatives) did not explain the residual heterogeneity (see Figure 5).

#### 3.3.2. BSFS Score

**Figure 6:**
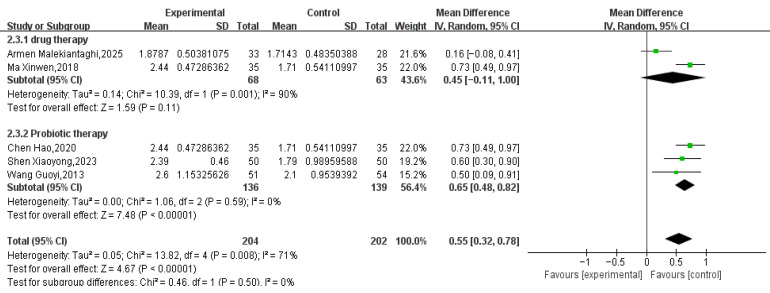
BSFS Scores: Tuina Plus Conventional Therapy vs. Conventional Therapy Alone—Subgroup Analysis by Control Type [28,33,35,40,41].

**Figure 7:**
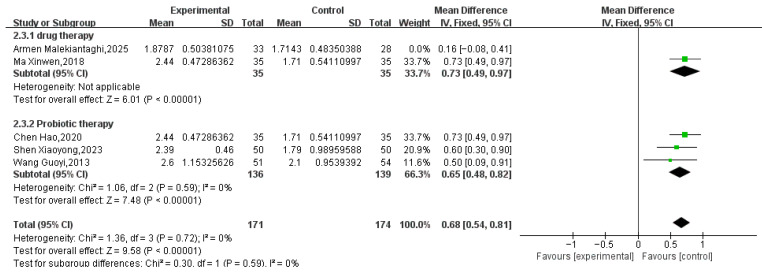
Adjusted for Detected Heterogeneity [28,33,35,40,41].

Five RCTs involving 406 pediatric patients (204 in the treatment group, 202 in the control group) reported BSFS scores. Meta-analysis showed that Tuina combined with conventional treatment significantly improved BSFS scores compared to conventional treatment alone (MD = 0.55, 95% CI 0.32 to 0.78, *p* < 0.00001; Tau^2^ = 0.05). However, substantial heterogeneity was detected (I^2^ = 71%; see Figure 6). The 95% prediction interval for the overall analysis ranged from −0.25 to 1.35, which crossed the null value (MD = 0), indicating that the true effect may vary substantially across future study settings. A sensitivity analysis excluding one study [33] eliminated the heterogeneity (I^2^ = 0% see Figure 7), and the pooled effect size remained stable (MD = 0.68, 95% CI 0.54 to 0.81, *p* < 0.00001).

#### 3.3.3. Recurrence Rate

**Figure 8:**
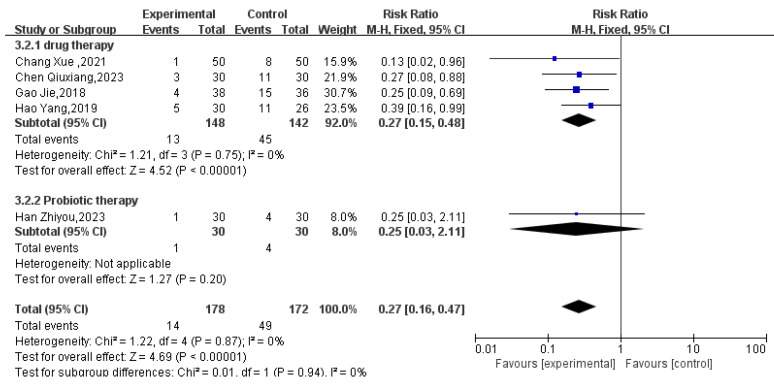
Comparison of Recurrence Rates: Massage-Adjunct versus Standard Therapy [31,34,36,37,38].

Five RCTs with efficacy data were included, involving 350 pediatric patients: 178 in the treatment group and 172 in the control group. Subgroup analysis was performed due to differing treatment modalities. Meta-analysis results showed that compared with conventional therapy alone, massage-assisted therapy for pediatric FC resulted in a lower recurrence rate than conventional therapy alone (relative risk RR = 0.27, 95% confidence interval [0.16, 0.47], *p* < 0.00001). Heterogeneity was minimal (I^2^ = 0%), indicating no significant heterogeneity (see Figure 8).

#### 3.3.4. Stool Difficulty Score

**Figure 9:**
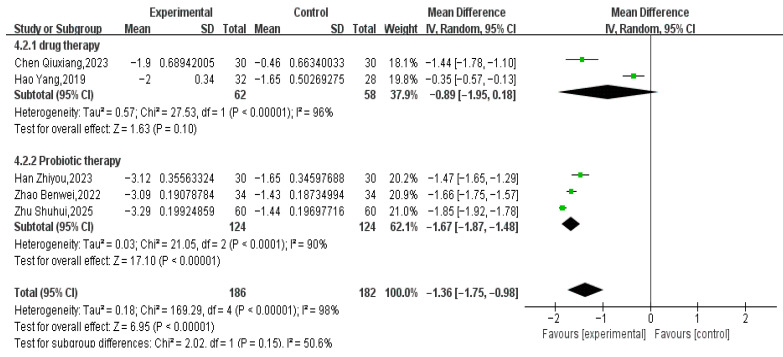
Comparison of Straining: Massage Adjuvant Therapy vs. Conventional Treatment [30,34,36,38,42].

Five RCTs involving 368 pediatric patients (186 in the treatment group and 182 in the control group) reported defecation difficulty scores. Meta-analysis showed that Tuina combined with conventional treatment significantly improved defecation difficulty scores compared to conventional treatment alone (MD = −1.36, 95% CI −1.75 to −0.98, *p* < 0.00001; Tau^2^ = 0.18). However, high heterogeneity was evident in both subgroups: I^2^ = 96% in the medication + massage group and I^2^ = 90% in the probiotics + massage group (see Figure 9). The 95% prediction interval for the overall analysis ranged from −2.85 to 0.13, which crossed the null value (MD = 0), indicating that the true effect may vary substantially across future study settings and could potentially be null or even harmful. Leave-one-out sensitivity analysis did not identify any single study as the primary source of this heterogeneity, and subgroup analysis based on type of control did not explain the variability.

#### 3.3.5. Adverse Reactions

Three studies reported adverse reactions. Among them, Chang Xue et al. (2021) [31], documented 2 adverse reactions in the control group versus 0 in the treatment group (*p* > 0.05), showing no statistical significance. However, the specific types of adverse reactions were not specified. Li Ying, 2019 [32], reported three adverse reactions in the treatment group and four in the control group (*p* = 0.6947), showing no statistical significance. The types of adverse reactions included mental fatigue, poor sleep, and decreased appetite. Zhu Shuhui et al. (2025) [30] reported 8 adverse reactions in the control group versus 0 in the treatment group (*p* = 0.003), which was statistically significant. The primary adverse reaction types were consistent with Li Ying’s report. Due to the limited number of studies reporting adverse reactions, it remains unclear whether massage therapy can mitigate side effects induced by control group treatments.

#### 3.3.6. Weekly Bowel Movement Frequency

Two RCTs involving 205 pediatric patients reported improvements in weekly bowel movement frequency. Each treatment group comprised 101 patients, while the control group had 104 participants. Shen Xiaoyong et al. (2023) [40] reported that after one month of treatment, weekly bowel movement frequency increased by 3.47 ± 0.757 compared to baseline, demonstrating a statistically significant difference versus the control group (*p* < 0.05). Wang Guoyi et al. (2013) [41] reported an increase of 2.6 ± 1.153 weekly bowel movements compared to the first week of treatment, which was statistically significant (*p* < 0.05) relative to the control group. Both studies indicated an increase in weekly bowel movements in the treatment group compared to the control group. However, due to the limited number of studies, definitive conclusions cannot be drawn.

#### 3.3.7. Assessment of Stability and Publication Bias for Primary Outcomes

**Figure 10:**
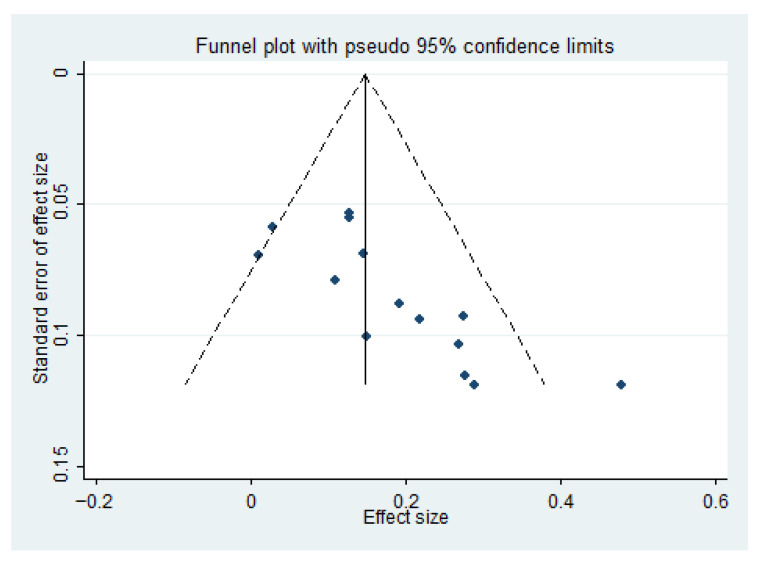
Funnel Plot for Efficacy.

**Figure 11:**
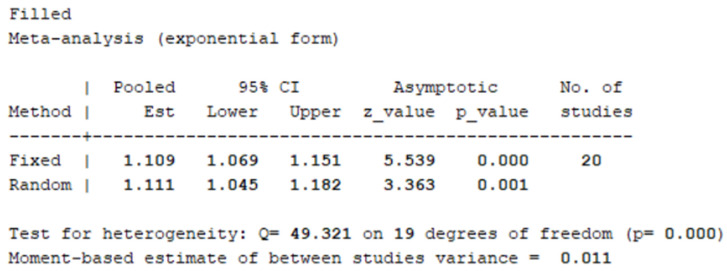
Results After Applying the Trim and Fill Method.

Publication bias was assessed using Stata 13. The funnel plot from 14 efficacy studies showed asymmetry and scatter (Figure 10), suggesting potential bias. The Egger’s test was used to assess publication bias. Results showed that the intercept term (bias coefficient) was 3.599 (95% CI: 1.639–5.559, *p* = 0.002), significantly deviating from zero. This indicates a small-sample effect, suggesting the potential presence of publication bias. The trim-and-fill method imputed 6 studies, yielding an adjusted effect size of RR = 1.109 (95% CI: 1.069, 1.151) (Figure 11), indicating a slightly strengthened effect.

**Figure 12:**
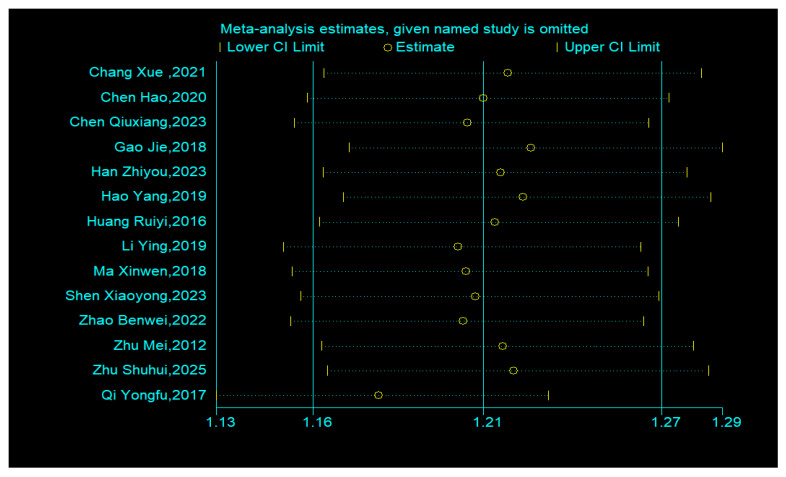
Sensitivity Analysis for Efficacy [28,29,30,31,32,34,35,36,37,38,39,40,42,43].

Sensitivity analysis revealed that the study by Qi et al., 2017 [29] notably influenced the overall results (Figure 12). For BSFS scores, due to the limited number of studies, only sensitivity analysis was performed, which identified two influential studies [33,41]. Given the small total number, the stability of this outcome is low.

## 4. Discussion

A total of 16 randomized controlled trials (RCTs) involving 1387 pediatric patients were included. The meta-analysis showed that, compared with conventional treatment alone, tuina combined with conventional treatment significantly improved the overall clinical response rate (RR = 1.18, 95% CI 1.10–1.25, *p* < 0.00001; Tau^2^ = 0.01), improved the Bristol Stool Form Scale (BSFS) score (MD = 0.55, 95% CI 0.32 to 0.78, *p* < 0.00001; Tau^2^ = 0.05), and reduced the defecation difficulty score (MD = −1.36, 95% CI −1.75 to −0.98, *p* < 0.00001; Tau^2^ = 0.18). However, there was significant heterogeneity among these outcome measures (I^2^ = 75%, 71%, and 96%, respectively). The 95% prediction intervals from the meta-analysis crossed the null value for all three primary outcome measures: treatment success rate (0.95–1.47; null RR = 1.0), BSFS score (−0.25 to 1.35; null MD = 0), and defecation difficulty score (−2.85 to 0.13; null MD = 0), suggesting that the true effect may vary significantly in future research settings and could even be null or have an adverse effect. Regarding recurrence, the relapse rate was lower with tuina-assisted treatment compared to conventional treatment alone (RR = 0.27, 95% CI 0.16–0.47, *p* < 0.00001). Although there were reports of improved weekly bowel movement frequency, this finding could not be confirmed due to insufficient sample size. It remains unclear whether tuina can alleviate treatment-related adverse reactions in the control group.

The pathogenesis of functional constipation in children is complex and multifactorial. Beyond physiological mechanisms, behavioral factors play a critical role. Incorrect toilet training, pain-induced defecation inhibition due to hard stools, and frequent rectal enemas can exacerbate fear and negative experiences associated with bowel movements, leading to intentional or unintentional fecal retention [5]. Children’s fear of defecation pain increases their anxiety levels, which may generalize to other situations and become a core negative psychological factor, further intensifying defecation difficulties [44]. These behaviors promote fecal retention in the rectum, where the mucosa absorbs water, resulting in harder stools and creating a vicious cycle that is difficult to break.

In clinical practice, the most commonly used first-line osmotic laxatives are polyethylene glycol (PEG) and lactulose [45]. Some studies report that PEG is more effective than lactulose in increasing stool frequency [46,47]. Regarding side effects, a ten-year survey revealed that children using PEG 3350 reported 1564 adverse symptoms. Among these adverse events, 58.75% were neurological or neuropsychiatric in nature, such as anxiety, irritability, and abnormal behavior [48]. However, this study has certain limitations. Lactulose has relatively fewer reported adverse reactions in pediatric use, with abdominal bloating being the most common [49]. A survey of 1066 healthcare professionals found that 70.6% considered probiotics appropriate for constipation [50]. Research has suggested that gut microbiota imbalance may contribute to functional constipation [51]. In an RCT, probiotics significantly improved stool frequency and fecal incontinence compared to placebo in pediatric FC, though they did not significantly enhance treatment success rates or alleviate symptoms like defecation pain and abdominal discomfort [52]. Another RCT for elderly FC patients showed that a multi-strain probiotic formulation significantly improved stool frequency and consistency over 12 weeks compared to placebo. Long-term probiotic use demonstrated cumulative effects, particularly evident in participants receiving probiotics beyond day 71 of treatment [53]. Given the diversity of probiotic strains, further investigation is needed to identify specific strains, dosages, and treatment durations most effective for constipation. Exploring potential probiotic-diet interactions and inter-individual variability is crucial to understanding response differences [54,55].

This study specifically investigates the role of tuina as an adjunctive intervention for childhood functional constipation. Its unique contribution, in the context of prior research, is delineated in the comparative summary presented in Table 3. Tuina, as a non-invasive therapy, is more suitable for young children, demonstrating strong tolerability and better long-term treatment adherence. This study provides a more comprehensive and systematic evaluation of Tuina-assisted treatment for pediatric FC through multiple outcome measures, offering richer and more robust evidence from an evidence-based medicine perspective.

However, several factors contribute to the uncertainty of the conclusions; to understand these factors, it is best to analyze them by examining their impact on specific outcomes.

Regarding treatment success rates, significant heterogeneity (I^2^ = 75%) was observed; after excluding one study [29], heterogeneity decreased to I^2^ = 57%, and the prediction interval no longer crossed the null value (RR = 1.0). It is possible that this study may have overestimated the treatment effect.

Regarding the BSFS score, heterogeneity (I^2^ = 71%) may be attributed to ethnic differences (all studies were conducted in Chinese or Iranian populations, which may have differing baseline bowel habits) and variations in massage techniques. Notably, one study [33] used Swedish massage rather than traditional Chinese tuina; after excluding this study, heterogeneity was completely eliminated (I^2^ = 0%).

Regarding the defecation difficulty score, although the highest heterogeneity was observed (I^2^ = 96%), neither sensitivity analysis nor subgroup analysis identified a single source. This may reflect broader clinical heterogeneity—including differences in outcome measurement tools, patient populations, and intervention protocols—which cannot be resolved by the available data.

In addition to the aforementioned statistical factors, the methodological quality of the included RCTs also undermined the reliability of the findings. Eleven studies did not explicitly describe the method used to generate the random sequence, which may have introduced selection bias. Furthermore, none of the RCTs employed a double-blind design. Given that this analysis compared tuina as an adjunctive treatment for functional constipation in children, blinding the therapists was impractical, and blinding the patients was also quite challenging. This may have introduced performance and measurement biases. Most outcome measures are susceptible to subjective factors, constituting another potential source of bias. Given that the majority of outcome measures in this review are subjective (e.g., overall efficacy, defecation difficulty scores), the lack of blinding is particularly problematic, as it may have systematically overestimated treatment effects. Funnel plot analysis indicated the presence of publication bias, which likely overestimated the efficacy of the intervention group. Furthermore, the literature search covered only Chinese and English databases, potentially omitting relevant studies published in other languages and thereby introducing language bias. Taken together, these biases may have led to an overestimation of the efficacy of Tuina.

Regarding external validity, the included studies originated solely from China and Iran; the resulting geographical limitations may affect the generalizability of the findings to other populations and healthcare settings. Significant variations in treatment duration (ranging from 10 days to 8 weeks) and follow-up periods across studies hindered the assessment of the long-term efficacy of Tuina. As a chronic condition, the treatment duration for functional constipation in most studies was less than one month; short-term improvements may not necessarily translate into sustained benefits. Furthermore, given the nature of Tuina interventions, achieving strict blinding poses inherent challenges.

Importantly, the reliance on overall effectiveness as a primary outcome measure warrants careful evaluation. This clinician-assessed composite measure is inherently subjective, and its definition varies slightly across studies. Coupled with the lack of blinded designs, this may lead to an overestimation of treatment effects and reduce the comparability of pooled results.

Overall, these findings suggest that when used as an adjunct to standard care, tuina may provide short-term symptom relief for functional constipation in children. However, given the substantial unexplained heterogeneity (particularly regarding the defecation difficulty score), the wide prediction intervals that crossed the null value for all three primary outcomes, and the methodological limitations, the pooled estimates should be considered exploratory rather than definitive. The current evidence is insufficient to support definitive clinical recommendations. There is an urgent need for large-scale, high-quality international multicenter studies with long follow-up periods, standardized and objective outcome measures, and strict blinding protocols to validate the findings of existing studies.

## 5. Implications for Future Research and Clinical Practice

This review highlights the following key areas for future research:

(1) At the methodological level, subsequent trials should implement strict randomization, allocation concealment, and blinded outcome assessment to minimize bias. Given the characteristics of tuina, employing sham-controlled designs or control groups with varying treatment durations may help isolate specific therapeutic effects. Standardized treatment protocols and extended follow-up periods (≥12 months) are needed to evaluate long-term efficacy and recurrence rates.
(2) Geographic limitations—studies concentrated in China and Iran—restrict the generalizability of conclusions; large-scale international multicenter trials are urgently needed. Prospective trial registration and publication of negative results should be encouraged to mitigate publication bias.
(3) In clinical practice, massage offers a non-invasive, well-tolerated adjunctive therapy for pediatric functional constipation, potentially reducing long-term laxative use. Its favorable safety profile supports application in young children and patients with poor medication tolerance.
(4) From a societal perspective, integrating evidence-based massage therapy into routine care can reduce healthcare costs by decreasing outpatient visits and medication-related adverse events. Future efforts should focus on developing standardized practitioner training programs and home education materials to facilitate the translation of research findings into clinical practice.

## 6. Conclusions

Existing evidence suggests that tuina, when used as an adjunct to conventional treatment, may improve treatment success rates, stool consistency (BSFS), and defecation difficulties in children with functional constipation, while also reducing recurrence rates. However, these findings should be interpreted with caution due to substantial heterogeneity across studies (I^2^ up to 96%), wide prediction intervals that crossed the null value for all three primary outcomes, methodological limitations in the included research (such as inadequate reporting of randomization and lack of blinding), and potential publication bias. Evidence regarding weekly stool frequency and adverse reactions remains unclear due to limited and inconsistent reporting. Furthermore, conclusions regarding long-term efficacy cannot be drawn due to short follow-up periods (most ≤2 months). The geographical concentration of studies (China and Iran) also limits generalizability to other populations. Large-scale, high-quality, international multicenter studies with extended follow-up periods are urgently needed to validate existing findings and establish the role of tuina in managing functional constipation in children.

## Figures and Tables

**Table 1:** The general characteristics of the 16 trials.

Name	Sample Size	Sex (Male/Female)		Age (Years), Mean ± SD		Intervention		Duration of Use	Outcome
	C/T	C	T	C	T	C	T		
Armen Malekiantaghi, 2025 [33]	28/31	17/11	18/14	6.36 ± 1.75	6.43 ± 1.81	drug therapy	massage + drug therapy	6 weeks	BSFS, CAS, Monitor the frequency of fecal incontinence, time to initiate defecation, straining during defecation, pain during defecation, and fecal withholding behavior
Chang Xue, 2021 [31]	50/50	26/24	28/22	3.25 ± 1.08	3.23 ± 1.06	drug therapy	massage + drug therapy	10 days	Overall Effectiveness, Constipation Symptom Score, Recurrence Rate, Adverse Reactions
Chen Hao, 2020 [35]	42/42	25/17	24/18	9.88 ± 0.34	9.57 ± 0.49	Medilac-Vita	massage + Medilac-Vita	2 weeks	BSFS, Overall Effectiveness
Chen Qiuxiang, 2023 [36]	30/30	16/14	18/12	5.13 ± 1.62	5.62 ± 1.94	Lactulose	massage + Lactulose	1 month	Overall Effectiveness, Constipation Symptom Score, Recurrence Rate
Gao Jie, 2018 [37]	39/40	21/18	23/17	3.2 ± 1. 4	5.4 ± 2. 1	Lactulose	massage + Lactulose	2 weeks	Overall Effectiveness; Recurrence Rate
Han Zhiyou, 2023 [38]	30/30	17/13	16/14	5.34 ± 0.86	5.42 ± 0.79	Bifidobacterium triple viable powder	massage + Bifidobacterium triple viable powder	1 month	Constipation Symptom Score, Overall Effectiveness, Wexner Constipation Score, Recurrence Rate
Hao Yang, 2019 [34]	28/32	13/15	12/20	3.43 ± 1.8	2.81 ± 1.71	Lactulose	massage + Lactulose	10 days	Constipation Symptom Score, Overall Effectiveness, Recurrence Rate
Huang Ruiyi, 2016 [39]	40/40	21/19	22/18	1.01 ± 0.94	1.80 ± 1.07	drug therapy	massage + drug therapy	2 months	Overall Effectiveness
Li Ying, 2019 [32]	48/48	27/21	28/20	4.08 ± 1.21	4.02 ± 1.25	Lactulose	massage + Lactulose	4 weeks	Overall Effectiveness, TCM syndrome score, Adverse Reactions
Ma Xinwen, 2018 [28]	35/35	19/16	21/14	2.1 ± 0.7	2.0 ± 0.6	Lactulose	massage + Lactulose	Not specified	Overall Effectiveness; Comparison of Spontaneous Bowel Movements, BSFS
Qi Yongfu, 2017 [29]	60/70	29/31	32/38	5.88 ± 3.91	6.29 ±3.06	Lactulose	massage + Lactulose	Not specified	Overall Effectiveness, Constipation Symptom Score, Recurrence Rate
Shen Xiaoyong, 2023 [40]	50/50	28/22	27/23	4.17 ± 0.56	4.14 ± 0.55	Live Combined Bifidobacterium and Lactobacillus Tablets	massage + Live Combined Bifidobacterium and Lactobacillus Tablets	1 month	Overall Effectiveness, BSFS, Weekly Bowel Movement Frequency
Wang Guoyi, 2013 [41]	54/51	Not specified	Not specified	Not specified	Not specified	Bifidobacterium triple viable capsules	massage + Bifidobacterium triple viable capsules	1 month	BSFS; Weekly Bowel Movement Frequency
Zhao Benwei, 2022 [42]	34/34	24/10	25/9	6.21 ± 1.02	6.01 ± 1.21	Bifidobacterium triple viable powder	massage + Bifidobacterium triple viable powder	1 month	Overall Effectiveness, Constipation Symptom Score
Zhu Mei, 2012 [43]	56/60	30/26	32/28	Not specified	Not specified	Bifidobacterium quadruple viable tablets	massage + Bifidobacterium quadruple viable tablets	1 month	Overall Effectiveness
Zhu Shuhui, 2025 [30]	60/60	27/33	26/34	6.18 ± 2.31	6.52 ± 2.44	Medilac-Vita	massage + Medilac-Vita	Not specified	Overall Effectiveness, TCM syndrome score, Recurrence Rate

Note: C: control group, T: treatment group, TCM: Traditional Chinese Medicine, CAS: Constipation Assessment Scale, BSFS: Bristol Stool Form Scale, Medilac-Vita: A probiotic preparation containing *Bacillus subtilis* and *Enterococcus faecium*.

**Table 2:** GRADE Summary of Findings for Main Outcomes.

Certainty Assessment							№ of Patients		Effect		Certainty	Importance
№ of Studies	Study Design	Risk of Bias	Inconsistency	Indirectness	Imprecision	Other Considerations	Efficacy	Placebo	Relative (95% CI)	Absolute (95% CI)		
Efficacy												
13	randomised trials	serious ^a^	not serious	not serious	not serious	none	527/551 (95.6%)	442/542 (81.5%)	RR 1.15 (1.09 to 1.21)	122 more per 1000 (from 73 more to 171 more)	⨁⨁⨁◯ Moderate ^a^	CRITICAL
BSFS Score												
5	randomised trials	serious ^b^	not serious	not serious	not serious	none	204	202	-	MD 0.55 higher (0.43 higher to 0.68 higher)	⨁⨁⨁◯ Moderate ^b^	CRITICAL

CI: Confidence interval; MD: mean difference; RR: risk ratio. ⨁◯: Grade of evidence (⨁⨁⨁⨁ high, ⨁⨁⨁◯ moderate, ⨁⨁◯◯ low, ⨁◯◯◯ very low). Explanations: ^a^ The outcome measurement is subjective and susceptible to performance bias and detection bias. ^b^ Although the BSFS is a standardized scale, its assessment in the included trials was not blinded and relies on patient recall/reporting, which introduces a risk of performance and detection bias.

**Table 3:** Comparison of Characteristics between the Current Study and Previously Published Systematic Reviews.

Study	Population	Key Distinctions of the Current Study
Current Study	Children	——
Gu X. et al. [56].	Adults	Different population (adults vs. children).
Wegh C.A.M. et al. [57].	Children	Different scope (broad overview of multiple interventions vs. specific focus on tuina) & different methodology (narrative review without meta-analysis vs. quantitative meta-analysis).
Yan L. et al. [58].	Children+ Adults	Different intervention model (tuina as monotherapy vs. tuina as adjunctive therapy to conventional care).
Li M. et al. [59].	Children with cerebral palsy	Different disease type (organic secondary constipation in special needs population vs. functional constipation in general pediatric population).
Fang Y.P. et al. [60].	Children	Different intervention model (tuina as monotherapy vs. tuina as adjunctive therapy).
Torres-Sánchez I. et al. [61].	Adults	Different population (adults vs. children).
Tang Y. et al. [62].	Adults	The study subjects and publication types differ (adults versus children, studies without results versus completed systematic reviews that include meta-analyses).
Bu F.L. et al. [63].	Children+ Adults	Different condition (IBS, which involves different diagnostic criteria and pathophysiology, vs. functional constipation).
Du W.F. et al. [64].	Children+ Adults	Different intervention (acupuncture vs. tuina).
Wang C. et al. [65].	Children+ Adults	Different publication type (study protocol without results vs. completed systematic review with meta-analysis).
Liu Z. et al. [66].	Children	More comprehensive search strategy (current study searched 8 databases including PubMed, Embase, Cochrane, Web of Science, CNKI, Wanfang, VIP, and CBM vs. only 4 databases in Liu et al., reducing risk of publication bias).

## Data Availability

No new data were created or analyzed in this study. Data sharing is not applicable to this article.

## Supplementary Materials

The following supporting information can be downloaded at: https://www.mdpi.com/article/10.3390/healthcare14070901/s1, File S1: Session Results.

## Abbreviations

RCTs	Randomized controlled trials
FC	Functional constipation
CNKI	China National Knowledge Infrastructure
CBM	China Biomedical Database
C	Control group
BSFS	Bristol Stool Form Scale
T	treatment group
TCM	Traditional Chinese Medicine
CAS	Constipation Assessment Scale
RR	relative risk
CI	confidence interval
MD	mean difference
SMD	standardized mean difference
