# Supplementary material for: Systematic Review and Meta-Analysis of Tuina as an Adjuvant Therapy for Functional Constipation in Children
Source: Healthcare (Basel). 2026 Mar 31;14(7):901. doi: 10.3390/healthcare14070901 (PMC13073099; doi:10.3390/healthcare14070901)
Supplement: Supplementary file 1 [file healthcare-14-00901-s001.zip › File S1.pdf]

Embase

Session Results

....

No.	Query Results	Results	Date
#17.	#12 AND #13 AND #16	126	21 Jan 2026
#16.	#14 OR #15	39,513	21 Jan 2026
#15.	massage	34,434	21 Jan 2026
#14.	tuina	5,578	21 Jan 2026
#13.	#6 OR #7 OR #8 OR #9 OR #10 OR #11	5,880,600	21 Jan 2026
#12.	#1 OR #2 OR #3 OR #5	138,004	21 Jan 2026
#11.	youngster	658	21 Jan 2026
#10.	kid	8,460	21 Jan 2026
#9.	children	2,791,220	21 Jan 2026
#8.	child	3,680,930	21 Jan 2026
#7.	adolescent	2,352,656	21 Jan 2026
#6.	pediatric	1,523,522	21 Jan 2026
#5.	dyschezia	1,094	21 Jan 2026
#4.	colonic AND inertia	408	21 Jan

2026

#3. colonic AND inertia 408 21 Jan 2026

#2. functional AND constipation 15,672 21 Jan 2026

#1. 'constipation'/exp 136,455 21 Jan 2026

PubMed

(((((Constipation) OR (Functional constipation)) OR (Colonic Inertia)) OR (Dyschezia)) AND ((((((pediatric) OR (adolescent)) OR (child)) OR (children)) OR (kid)) OR (youngster))) AND ((Tuina) OR (massage))
(Tuina) OR (massage)
massage
Tuina
(((((pediatric) OR (adolescent)) OR (child)) OR (children)) OR (kid)) OR (youngster)
youngster
kid
children
child
adolescent
pediatric
(((Constipation) OR (Functional constipation)) OR (Colonic Inertia)) OR (Dyschezia)
Dyschezia
Colonic Inertia
Functional constipation
Constipation

Web of Science

#16 (((ALL=(Functional constipation)) OR ALL=(Constipation)) OR ALL=(Colonic Inertia)) OR ALL=(Dyschezia)
#18 (((((ALL=(pediatric)) OR ALL=(adolescent)) OR ALL=(child)) OR ALL=(children)) OR ALL=(kid)) OR ALL=(youngster)
#24 (ALL=(Tuina)) OR ALL=(massage)
#24 AND #18 AND #16

the Cochrane Library
#1 MeSH descriptor: [Constipation] explode all trees 2508
#2 (Functional constipation):ti,ab,kw (Word variations have been searched) 4695
#3 (Colonic Inertia):ti,ab,kw (Word variations have been searched) 19
#4 (Dyschezia):ti,ab,kw (Word variations have been searched) 90
#5 #4 OR #3 OR #2 OR #1 6343
#6 (pediatric):ti,ab,kw (Word variations have been searched) 51579
#7 (adolescent):ti,ab,kw (Word variations have been searched) 181434
#8 (child):ti,ab,kw (Word variations have been searched) 205561
#9 (children):ti,ab,kw (Word variations have been searched) 205561
#10 (kid):ti,ab,kw (Word variations have been searched) 1984
#11 (youngster):ti,ab,kw (Word variations have been searched) 213
#12 #6 OR #7 OR #8 OR #9 OR #10 OR #11 333556
#13 (Tuina):ti,ab,kw (Word variations have been searched) 399
#14 (massage):ti,ab,kw (Word variations have been searched) 9328
#15 #13 OR #14 9612
#16 #15 AND #12 AND #5 32

CNKI 21
(SU='小儿' OR SU='婴儿' OR SU='幼儿' OR SU='儿童' OR SU='婴幼儿' OR SU='kid' OR SU='child*' OR SU='infant*') AND (SU='便秘' OR SU='constipation' OR SU='功能性便秘') AND ((SU='推拿' OR SU='按摩' OR SU='手法' OR SU='按揉' OR SU='小儿推拿' OR SU='捏脊' OR SU='massage') AND (SU='乳果糖' OR SU='聚乙二醇' OR SU='开塞露' OR SU='西药' OR SU='常规治疗' OR SU='药物' SU='益生菌' OR SU='微生态制剂' OR SU='合生元')) AND (SU='对照' OR SU='对比' OR SU='比较' OR SU='vs' OR SU='疗效')

WanFang Database 83
(主题:("小儿" OR "婴儿" OR "幼儿" OR "儿童" OR "婴幼儿") AND 主题:("功能性便秘" OR "便秘")) AND (主题:("推拿" OR "按摩" OR "小儿推拿" OR "捏脊") AND 主题:("益生菌" OR "微生态制剂" OR "合生元" OR "乳果糖" OR "聚乙二醇" OR "开塞露" OR "西药" OR "常规治疗")) AND 主题:("对照" OR "对比" OR "比较" OR "vs" OR "疗效" OR "随机")

VIP32

(U=小儿 OR U=婴儿 OR U=幼儿 OR U=儿童) AND (U=功能性便秘 OR U=便秘) AND (U=推拿 OR U=按摩 OR U=小儿推拿) AND (U=益生菌 OR U=乳果糖 OR U=聚乙二醇 OR U=西药) AND (U=对照 OR U=对比 OR U=随机)

SinoMed (CBM) 39

("小儿"[常用字段:智能] OR "儿童"[常用字段:智能] OR "婴儿"[常用字段:智能]) AND ("功能性便秘"[常用字段:智能] OR "便秘"[常用字段:智能]) AND ("推拿"[常用字段:智能] OR "按摩"[常用字段:智能] OR "小儿推拿"[常用字段:智能]) AND ("益生菌"[常用字段:智能] OR "微生态制剂"[常用字段:智能] OR "乳果糖"[常用字段:智能] OR "聚乙二醇"[常用字段:智能] OR "西药"[常用字段:智能] OR "常规治疗"[常用字段:智能]) AND ("对照"[常用字段:智能] OR "对比"[常用字段:智能] OR "随机"[常用字段:智能])
